# Reply to Dr. W. H. Trueman

**Published:** 1895-09

**Authors:** I. Norman Broomell

**Affiliations:** Philadelphia


### REPLY TO DR. W. H. TRUEMAN.[1]

BY DR. I. NORMAN BROOMELL, PHILADELPHIA.

Mr. Chairman,—I ask a few minutes of your time in which to make a short reply to Dr. William H. Trueman's paper published in the December number of the International Dental Journal, p. 761. In his address the doctor took occasion to refer several times to a recent article read by myself before this Society (International Dental Journal, October, 1894, p. 620). As the paper which he criticises so emphatically originated in this Society, it would seem eminently proper for me to produce my reply here. I was extremely gratified when I found that Dr. Trueman had considered my article worthy of his notice. Criticisms from such an eminent professional brother are both to be respected and desired. Criticisms upon any subject will always give to it life and vigor, and it is by these interchanges of opinions that we prosper.

The subject of Dr. Trueman's paper compels me to believe that he intentionally or otherwise misconstrues the meaning of my article. Throughout his essay he strongly endeavors to have his hearers believe that I would have vulcanite rubber banished from the dental laboratory. This was not my meaning, and I expressed my ideas clearly and concisely when I said that it was not the use so much as the abuse of rubber that is working the damage. I can plainly see how the first paragraph of my paper might suggest to Dr. Trueman the question which he has used as a title to his article. The question, which he has both asked and answered,—"Is prosthetic dentistry lagging? No,"—was prompted by my assertion that while there had been great advances in all operations upon the natural teeth, there had not been a corresponding progress in prosthetic dentistry. The gentleman wanders far from his text, treating the whole subject as though I had said that prosthetic dentistry is retrograding. I am free to admit the wonderful advances in both branches of our calling, but am firmly impressed with the idea that plastics (blessings though they be to thousands) have forced dental prosthesis to the rear.

Dr. Trueman does not believe that vulcanite rubber is the father of the dental charlatan, and brands my suggestions upon this point as "absurd," "saddening," "humiliating," etc.

He attempts to strengthen his assertions by the statement that

---

[1] Read before the Odontological Society of Pennsylvania, January 12, 1895.

"writers of a century and a half ago speak as glibly of dental quacks" as do writers of the present day. How could it be possible for a profession to be invaded by pretenders or quacks when such a profession did not at that time exist. I quote from one of the very best authorities when I tell you that dentistry has only taken rank as a distinct profession within the last century. "From advertisements in the newspapers of 1803, the practice of making and cleaning teeth appears to have been in the hands of jewellers and silversmiths." With the "business" in so primitive a condition at the beginning of the present century, we can only refer to all those giving attention to it at that time as pretenders. The doctor would have all "who rose as exponents of dental thought" uncover the pages of history to find "the evidence through which scientific conclusions should be sought." My paper made no pretentions to science; it was intended to be a simple, plain, every-day, common-sense composition, written for the purpose of expressing the thoughts of the writer, without the slightest desire or intention to elaborate or deceive, by the introduction of spread-eagle paragraphs, frequently as deep and as bottomless as the ocean, as dry as the desert, lines calculated to bring slumber to the most chronic case of insomnia. My friend speaks of the long and tedious days spent upon the finishing of a gold or silver denture, of the skill required and time expended in refining and preparing the metals; of the bending of plates into shape, and quite likely their horrible mutilation by that fiendish tool the horn mallet. We are living in an age of advancement. To-day gold plate is as readily obtained as vulcanite rubber, horn mallets will soon be a thing of the past, and when a metal plate is brought into shape by the modern method, there is no necessity of the weary days of "rubbing, rubbing, rubbing."

With all these changes, so much greater the shame that gold plates are not more frequently recommended. What Dr. Trueman condemns in one paragraph he admits in the next; his admissions amounting to little less than a complete acceptance of my ideas, which, as he says, were written without thought, while from his pen flowed the more thoughtful thoughts of a "better informed and thoughtful man."

In conclusion, Mr. President, I must thank Dr. Trueman for his one word of praise.

My paper, while possessing no good qualities, emanating from the "expressions learned parrot-like," which are among the "stock fads" constantly used in our dental meetings, and very much like the "stuff" in all recent reports of society meetings; this paper, with ideas so "absurd" and amusing, both "saddening" and "humiliating" to him, receives the compliment of being fully up to the times, and on an equal with the general literature of to day. Whither are we drifting?

Such expressions as the foregoing quotations, while probably interesting and entertaining to the writer, can hardly be considered within the code of ethics, and show upon their face a lack of good judgment. After all, which should be condemned, the writing or the publication of this class of criticism?

